# Bicyclo­[2.2.2]oct-7-ene-2,3,5,6-tetra­carboxylic dianhydride

**DOI:** 10.1107/S1600536808012452

**Published:** 2008-05-10

**Authors:** Tuoping Hu

**Affiliations:** a Department of Chemistry, North University of China, Taiyuan, Shanxi 030051, People’s Republic of China

## Abstract

In the title compound, C_12_H_8_O_6_, mol­ecules are linked by weak C—H⋯O inter­actions involving all the potential donors, generating a three-dimensional network.
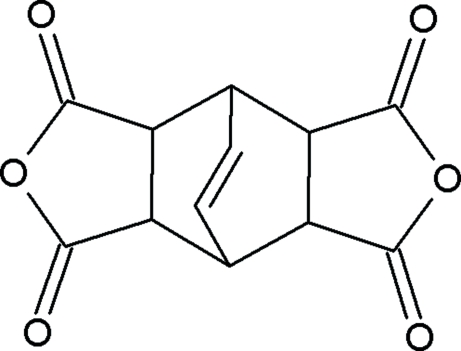

## Experimental

### 

#### Crystal data

C_12_H_8_O_6_
*M* *_r_* = 248.18
Monoclinic, 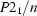

*a* = 7.627 (2) Å
*b* = 13.877 (3) Å
*c* = 9.823 (2) Å
β = 100.68 (2)°
*V* = 1021.7 (4) Å^3^
*Z* = 4
Mo *K*α radiation
μ = 0.13 mm^−1^
*T* = 296 (2) K
0.28 × 0.16 × 0.10 mm

#### Data collection

Bruker SMART CCD area-detector diffractometer
Absorption correction: multi-scan (*SADABS*; Sheldrick, 1996 ▶) *T*_min_ = 0.921, *T*_max_ = 0.987
11412 measured reflections
2119 independent reflections
1460 reflections with *I* > 2σ(*I*)
*R*_int_ = 0.051

#### Refinement

*R*[*F* ^2^ > 2σ(*F* ^2^)] = 0.042
*wR*(*F* ^2^) = 0.099
*S* = 1.01
2118 reflections
164 parameters
H-atom parameters constrained
Δρ_max_ = 0.21 e Å^−3^
Δρ_min_ = −0.16 e Å^−3^

### 

Data collection: *SMART* (Bruker, 2007 ▶); cell refinement: *SAINT-Plus* (Bruker, 2007 ▶); data reduction: *SAINT-Plus*; program(s) used to solve structure: *SHELXS97* (Sheldrick, 2008 ▶); program(s) used to refine structure: *SHELXL97* (Sheldrick, 2008 ▶); molecular graphics: *SHELXTL* (Sheldrick, 2008 ▶); software used to prepare material for publication: *SHELXTL*.

## Supplementary Material

Crystal structure: contains datablocks global, I. DOI: 10.1107/S1600536808012452/sg2233sup1.cif

Structure factors: contains datablocks I. DOI: 10.1107/S1600536808012452/sg2233Isup2.hkl

Additional supplementary materials:  crystallographic information; 3D view; checkCIF report

## Figures and Tables

**Table 1:** Hydrogen-bond geometry (Å, °)

*D*—H⋯*A*	*D*—H	H⋯*A*	*D*⋯*A*	*D*—H⋯*A*
C3—H3⋯O2^i^	0.98	2.50	3.313 (3)	140
C8—H8⋯O3^ii^	0.98	2.58	3.175 (2)	119

Symmetry codes: (i) 
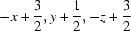
; (ii) 
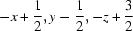
.

**Fig. 1.**
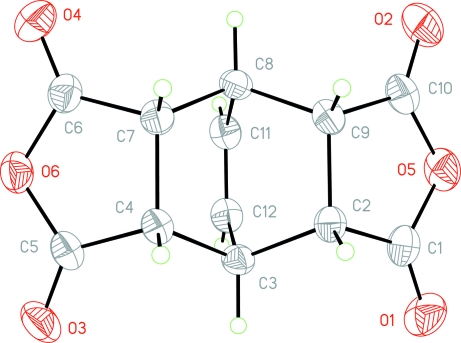
Molecular structure showing 50% probability displacement ellipsoids.

**Fig. 2.**
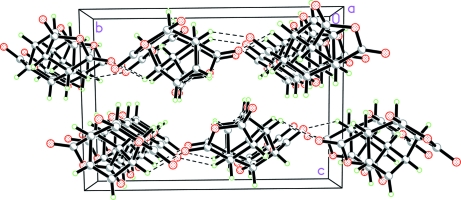
Packing diagram viewed down the a axis.

## supplementary crystallographic information

### Comment

The molecule of the title complex, (I) (Fig. 1), is neutral. Molecules are linked by C—H···O weak interactions involving all the potential donors, generating a three-dimensional network, as shown in Fig. 2. No conventional hydrogen bonding was found in the structure.

### Experimental

The title compound was obtained unintentionally as the product of an attempted synthesis of a polymeric network complex of znic with the bicyclo[2.2.2]oct-7-ene-2,3,5,6-tetracarboxylic acid. The title compound (0.4 mmol) and Zn(NO_3_)_2_. 6H_2_O (0.2 mmol) were dissolved in 15 ml distilled water, to which 2 drops of H_3_PO4 (w.t. 18%) was added. The solution was put into the oven at 50 centigrade degree for 1 day. Colourless prism crystals were collected by filtration.

### Refinement

H atoms were positioned geometrically and refined using a riding model with C—H = 0.980 Å and 0.930 Å, respectively, with *U*_iso_(H) = 1.2 times *U*_eq_(C). Reflection -111 was omitted because it was eclipsed by the beam stop.

### Crystal data

**Table:**

C_12_H_8_O_6_	*F*_000_ = 512
*M**_r_* = 248.18	*D*_x_ = 1.613 Mg m^−^^3^
Monoclinic, *P*2_1_/*n*	Mo *K*α radiation λ = 0.71073 Å
Hall symbol: -P 2yn	Cell parameters from 2677 reflections
*a* = 7.627 (2) Å	θ = 2.9–24.1º
*b* = 13.877 (3) Å	µ = 0.13 mm^−^^1^
*c* = 9.823 (2) Å	*T* = 296 (2) K
β = 100.68 (2)º	Prism, colourless
*V* = 1021.7 (4) Å^3^	0.28 × 0.16 × 0.10 mm
*Z* = 4	

### Data collection

**Table:**

Bruker SMART CCD area-detector diffractometer	2119 independent reflections
Radiation source: fine-focus sealed tube	1460 reflections with *I* > 2σ(*I*)
Monochromator: graphite	*R*_int_ = 0.051
*T* = 296(2) K	θ_max_ = 26.5º
φ and ω scans	θ_min_ = 2.6º
Absorption correction: multi-scan(SADABS; Sheldrick, 1996)	*h* = −9→9
*T*_min_ = 0.921, *T*_max_ = 0.987	*k* = −17→15
11412 measured reflections	*l* = −11→12

### Refinement

**Table:**

Refinement on *F*^2^	Hydrogen site location: inferred from neighbouring sites
Least-squares matrix: full	H-atom parameters constrained
*R*[*F*^2^ > 2σ(*F*^2^)] = 0.042	*w* = 1/[σ^2^(*F*_o_^2^) + (0.0334*P*)^2^ + 0.3914*P*] where *P* = (*F*_o_^2^ + 2*F*_c_^2^)/3
*wR*(*F*^2^) = 0.099	(Δ/σ)_max_ < 0.001
*S* = 1.01	Δρ_max_ = 0.21 e Å^−^^3^
2118 reflections	Δρ_min_ = −0.16 e Å^−^^3^
164 parameters	Extinction correction: SHELXL97 (Sheldrick, 2008), Fc^*^=kFc[1+0.001xFc^2^λ^3^/sin(2θ)]^-1/4^
Primary atom site location: structure-invariant direct methods	Extinction coefficient: 0.005 (1)
Secondary atom site location: difference Fourier map	

### Special details

**Table:**

Geometry. All e.s.d.'s (except the e.s.d. in the dihedral angle between two l.s. planes) are estimated using the full covariance matrix. The cell e.s.d.'s are taken into account individually in the estimation of e.s.d.'s in distances, angles and torsion angles; correlations between e.s.d.'s in cell parameters are only used when they are defined by crystal symmetry. An approximate (isotropic) treatment of cell e.s.d.'s is used for estimating e.s.d.'s involving l.s. planes.
Refinement. Refinement of *F*^2^ against ALL reflections. The weighted *R*-factor *wR* and goodness of fit *S* are based on *F*^2^, conventional *R*-factors *R* are based on *F*, with *F* set to zero for negative *F*^2^. The threshold expression of *F*^2^ > σ(*F*^2^) is used only for calculating *R*-factors(gt) *etc*. and is not relevant to the choice of reflections for refinement. *R*-factors based on *F*^2^ are statistically about twice as large as those based on *F*, and *R*- factors based on ALL data will be even larger.

### Fractional atomic coordinates and isotropic or equivalent isotropic displacement parameters (Å^2^)

**Table:**

	*x*	*y*	*z*	*U*_iso_*/*U*_eq_	
C1	0.7820 (3)	0.83700 (16)	0.6304 (2)	0.0449 (5)	
C2	0.5829 (2)	0.84372 (13)	0.61717 (19)	0.0355 (4)	
H2	0.5239	0.8381	0.5200	0.043*	
C3	0.5305 (2)	0.93909 (13)	0.68048 (19)	0.0350 (4)	
H3	0.5646	0.9952	0.6307	0.042*	
C4	0.3256 (2)	0.93415 (13)	0.67248 (18)	0.0344 (4)	
H4	0.2633	0.9301	0.5760	0.041*	
C5	0.2627 (3)	1.01999 (15)	0.7435 (2)	0.0416 (5)	
C6	0.1858 (3)	0.89126 (15)	0.8635 (2)	0.0434 (5)	
C7	0.2788 (2)	0.84782 (13)	0.75613 (18)	0.0352 (4)	
H7	0.1984	0.8039	0.6962	0.042*	
C8	0.4506 (2)	0.79445 (13)	0.82409 (18)	0.0354 (4)	
H8	0.4251	0.7413	0.8831	0.042*	
C9	0.5347 (2)	0.75817 (13)	0.70190 (19)	0.0358 (4)	
H9	0.4526	0.7145	0.6429	0.043*	
C10	0.7097 (3)	0.70890 (16)	0.7535 (2)	0.0462 (5)	
C11	0.5767 (2)	0.86683 (14)	0.90425 (19)	0.0392 (5)	
H11	0.6221	0.8602	0.9984	0.047*	
C12	0.6180 (2)	0.94061 (14)	0.83043 (19)	0.0394 (5)	
H12	0.6951	0.9895	0.8686	0.047*	
O1	0.88296 (19)	0.89053 (13)	0.58906 (17)	0.0638 (5)	
O2	0.7409 (2)	0.64134 (12)	0.82843 (18)	0.0681 (5)	
O3	0.2829 (2)	1.10382 (11)	0.72476 (16)	0.0596 (4)	
O4	0.1289 (2)	0.85316 (12)	0.95463 (16)	0.0629 (5)	
O5	0.84589 (17)	0.75576 (11)	0.70513 (15)	0.0537 (4)	
O6	0.17498 (18)	0.99043 (10)	0.84767 (14)	0.0485 (4)	

### Atomic displacement parameters (Å^2^)

**Table:**

	*U*^11^	*U*^22^	*U*^33^	*U*^12^	*U*^13^	*U*^23^
C1	0.0359 (11)	0.0528 (13)	0.0462 (12)	0.0033 (10)	0.0079 (9)	−0.0067 (10)
C2	0.0311 (9)	0.0378 (11)	0.0373 (10)	0.0016 (8)	0.0054 (7)	−0.0023 (8)
C3	0.0334 (9)	0.0298 (10)	0.0422 (10)	−0.0030 (8)	0.0076 (8)	0.0016 (8)
C4	0.0337 (9)	0.0331 (10)	0.0351 (9)	0.0039 (8)	0.0031 (7)	−0.0014 (8)
C5	0.0405 (11)	0.0389 (12)	0.0429 (11)	0.0098 (9)	0.0018 (9)	−0.0003 (9)
C6	0.0355 (10)	0.0470 (13)	0.0482 (12)	0.0008 (9)	0.0089 (9)	−0.0024 (10)
C7	0.0313 (9)	0.0327 (11)	0.0411 (10)	−0.0025 (8)	0.0052 (8)	−0.0035 (8)
C8	0.0375 (10)	0.0297 (10)	0.0390 (10)	0.0002 (8)	0.0070 (8)	0.0033 (8)
C9	0.0328 (9)	0.0309 (10)	0.0423 (10)	0.0017 (8)	0.0033 (8)	−0.0024 (8)
C10	0.0455 (12)	0.0419 (13)	0.0490 (12)	0.0112 (10)	0.0031 (9)	−0.0021 (10)
C11	0.0386 (10)	0.0415 (11)	0.0353 (10)	0.0047 (9)	0.0013 (8)	−0.0021 (9)
C12	0.0349 (10)	0.0369 (11)	0.0448 (11)	−0.0035 (8)	0.0034 (8)	−0.0078 (9)
O1	0.0392 (8)	0.0791 (12)	0.0768 (11)	−0.0069 (8)	0.0205 (8)	0.0052 (9)
O2	0.0689 (11)	0.0550 (10)	0.0784 (11)	0.0261 (8)	0.0083 (9)	0.0170 (9)
O3	0.0726 (11)	0.0345 (9)	0.0716 (11)	0.0137 (7)	0.0131 (8)	0.0022 (8)
O4	0.0655 (10)	0.0696 (11)	0.0616 (10)	−0.0019 (8)	0.0328 (8)	0.0038 (8)
O5	0.0326 (7)	0.0582 (10)	0.0685 (10)	0.0107 (7)	0.0044 (7)	0.0018 (8)
O6	0.0500 (8)	0.0464 (9)	0.0516 (8)	0.0096 (6)	0.0161 (7)	−0.0056 (7)

### Geometric parameters (Å, °)

**Table:**

C1—O1	1.194 (2)	C6—O6	1.386 (2)
C1—O5	1.384 (2)	C6—C7	1.502 (3)
C1—C2	1.503 (3)	C7—C8	1.545 (2)
C2—C9	1.533 (3)	C7—H7	0.9800
C2—C3	1.546 (3)	C8—C11	1.508 (3)
C2—H2	0.9800	C8—C9	1.546 (3)
C3—C12	1.500 (3)	C8—H8	0.9800
C3—C4	1.551 (2)	C9—C10	1.502 (3)
C3—H3	0.9800	C9—H9	0.9800
C4—C5	1.504 (3)	C10—O2	1.188 (2)
C4—C7	1.532 (3)	C10—O5	1.382 (3)
C4—H4	0.9800	C11—C12	1.326 (3)
C5—O3	1.192 (2)	C11—H11	0.9300
C5—O6	1.384 (2)	C12—H12	0.9300
C6—O4	1.189 (2)		
			
O1—C1—O5	120.12 (18)	C6—C7—C8	111.21 (15)
O1—C1—C2	129.6 (2)	C4—C7—C8	110.07 (15)
O5—C1—C2	110.29 (18)	C6—C7—H7	110.3
C1—C2—C9	104.20 (15)	C4—C7—H7	110.3
C1—C2—C3	110.54 (15)	C8—C7—H7	110.3
C9—C2—C3	109.79 (15)	C11—C8—C7	108.31 (15)
C1—C2—H2	110.7	C11—C8—C9	107.83 (15)
C9—C2—H2	110.7	C7—C8—C9	105.15 (14)
C3—C2—H2	110.7	C11—C8—H8	111.7
C12—C3—C2	107.81 (15)	C7—C8—H8	111.7
C12—C3—C4	108.10 (15)	C9—C8—H8	111.7
C2—C3—C4	105.96 (14)	C10—C9—C2	104.37 (15)
C12—C3—H3	111.6	C10—C9—C8	110.90 (15)
C2—C3—H3	111.6	C2—C9—C8	110.18 (14)
C4—C3—H3	111.6	C10—C9—H9	110.4
C5—C4—C7	104.15 (16)	C2—C9—H9	110.4
C5—C4—C3	110.29 (15)	C8—C9—H9	110.4
C7—C4—C3	109.89 (14)	O2—C10—O5	120.48 (18)
C5—C4—H4	110.8	O2—C10—C9	129.2 (2)
C7—C4—H4	110.8	O5—C10—C9	110.31 (17)
C3—C4—H4	110.8	C12—C11—C8	114.96 (16)
O3—C5—O6	119.82 (19)	C12—C11—H11	122.5
O3—C5—C4	129.8 (2)	C8—C11—H11	122.5
O6—C5—C4	110.36 (17)	C11—C12—C3	114.75 (17)
O4—C6—O6	120.26 (19)	C11—C12—H12	122.6
O4—C6—C7	129.5 (2)	C3—C12—H12	122.6
O6—C6—C7	110.22 (17)	C10—O5—C1	110.67 (15)
C6—C7—C4	104.49 (15)	C5—O6—C6	110.52 (16)
			
O1—C1—C2—C9	−175.3 (2)	C4—C7—C8—C9	−62.20 (17)
O5—C1—C2—C9	3.2 (2)	C1—C2—C9—C10	−1.16 (19)
O1—C1—C2—C3	−57.4 (3)	C3—C2—C9—C10	−119.57 (16)
O5—C1—C2—C3	121.13 (17)	C1—C2—C9—C8	117.95 (15)
C1—C2—C3—C12	−59.7 (2)	C3—C2—C9—C8	−0.46 (19)
C9—C2—C3—C12	54.68 (18)	C11—C8—C9—C10	61.6 (2)
C1—C2—C3—C4	−175.28 (15)	C7—C8—C9—C10	176.98 (15)
C9—C2—C3—C4	−60.87 (18)	C11—C8—C9—C2	−53.47 (18)
C12—C3—C4—C5	59.5 (2)	C7—C8—C9—C2	61.93 (17)
C2—C3—C4—C5	174.86 (15)	C2—C9—C10—O2	176.5 (2)
C12—C3—C4—C7	−54.75 (19)	C8—C9—C10—O2	57.9 (3)
C2—C3—C4—C7	60.60 (18)	C2—C9—C10—O5	−1.2 (2)
C7—C4—C5—O3	172.72 (19)	C8—C9—C10—O5	−119.86 (17)
C3—C4—C5—O3	54.9 (3)	C7—C8—C11—C12	−56.8 (2)
C7—C4—C5—O6	−4.85 (19)	C9—C8—C11—C12	56.5 (2)
C3—C4—C5—O6	−122.71 (16)	C8—C11—C12—C3	0.4 (2)
O4—C6—C7—C4	−177.8 (2)	C2—C3—C12—C11	−57.5 (2)
O6—C6—C7—C4	0.55 (19)	C4—C3—C12—C11	56.6 (2)
O4—C6—C7—C8	−59.1 (3)	O2—C10—O5—C1	−174.58 (19)
O6—C6—C7—C8	119.27 (16)	C9—C10—O5—C1	3.4 (2)
C5—C4—C7—C6	2.49 (18)	O1—C1—O5—C10	174.51 (19)
C3—C4—C7—C6	120.62 (15)	C2—C1—O5—C10	−4.2 (2)
C5—C4—C7—C8	−117.01 (16)	O3—C5—O6—C6	−172.38 (18)
C3—C4—C7—C8	1.1 (2)	C4—C5—O6—C6	5.5 (2)
C6—C7—C8—C11	−62.4 (2)	O4—C6—O6—C5	174.79 (18)
C4—C7—C8—C11	52.87 (19)	C7—C6—O6—C5	−3.7 (2)
C6—C7—C8—C9	−177.52 (15)		

### Hydrogen-bond geometry (Å, °)

**Table:**

*D*—H···*A*	*D*—H	H···*A*	*D*···*A*	*D*—H···*A*
C3—H3···O2^i^	0.98	2.50	3.313 (3)	140
C8—H8···O3^ii^	0.98	2.58	3.175 (2)	119

Symmetry codes: (i) −*x*+3/2, *y*+1/2, −*z*+3/2; (ii) −*x*+1/2, *y*−1/2, −*z*+3/2.
